# Supplementary material for: Dynamic transcriptomic profiles of zebrafish gills in response to zinc supplementation
Source: BMC Genomics. 2010 Oct 11;11:553. doi: 10.1186/1471-2164-11-553 (PMC3091702; doi:10.1186/1471-2164-11-553)
Supplement: Additional file 2 — Interactive Direct Interaction Network representing the molecular interactions between zinc, copper, iron, calcium and proteins encoded by transcripts changed by zinc supplementation. Mini web-site containing index.html and hyperlinked pages in subdirectory describing a Direct Interaction Network automatically generated based on curated interactions contained within the proprietary PathwayArchitect database. Ovals represent proteins and the circles symbolize metal ions. Objects are coloured by their abundance in zebrafish at the time-point they were significantly different from the control is a scale from -4 fold (dark green) to +4 fold (dark red). Where significant differences were found at more than one time-point, the colour overlay shows expression at the first instance. Dark blue squares denote 'binding', and light blue squares 'expression'; green squares stand for 'regulation', green diamonds for 'metabolism', and green circles for 'promoter binding'. Arrow heads indicate directionality of the interaction where annotated. All nodes and edges can be further interrogated by selecting the relative area of the image. [file 1471-2164-11-553-S2.zip › PathwayArchitect Zn xs DIN/666745.html]

# REGULATION:

|  |  |
| --- | --- |
| Type | REGULATION |
| Effect | Positive |


---

|  |  |
| --- | --- |
| Score | 0 |


---

|  |  |
| --- | --- |
| Reference Count | 4 |


---

|  |  |
| --- | --- |
| Mechanism | Unknown |


---

|  |  |
| --- | --- |
| Reference:0 || Sentence | "The iron-induced induction of FPN1 mRNA was blocked by actinomycin D, suggesting that transcriptional control was responsible for this effect." |
| PMID | 12907459 |
| Year | 2003 |
| Species | Mouse |
|  | Human |
|  | Rat |
| Journal | Blood |
| RefScore | 0 |
| Source | PArchNLP |
  |
|  |  |


---

|  |  |
| --- | --- |
 Reference:1 || Sentence | "Mutations in the gene encoding the iron exporter, ferroportin 1, cause iron overload characterized by iron accumulation in macrophages yet normal plasma iron levels." |
| PMID | 12382200 |
| Year | 2002 |
| Species | Human |
| Journal | Semin Hematol |
| RefScore | 0 |
| Source | PArchNLP |
  ||  |  |


---

|  |  |
| --- | --- |
 Reference:2 || Sentence | "Both iron-deficient wild-type mice and sla mice showed increased expression of Heph and Ireg1 mRNA, compared to controls, whereas only iron-deficient wild-type mice had increased expression of the brush border transporter Dmt1." |
| PMID | 12730111 |
| Year | 2003 |
| Species | Mouse |
| Journal | Blood |
| RefScore | 1 |
| Source | PArchNLP |
  ||  |  |


---

|  |  |
| --- | --- |
 Reference:3 || Sentence | However, iron had no effect on HAMP expression but was able to upregulate both DMT1 and FPN1 in alveolar macrophages. |
| PMID | 16648237 |
| Year | 2006 |
| Species | Mouse |
| Journal | Am J Physiol Lung Cell Mol Physiol |
| RefScore | 2 |
| Source | PArchNLP |
  |


---

|  |  |
| --- | --- |
